# Petri-plate, bacteria, and laser optical scattering sensor

**DOI:** 10.3389/fcimb.2022.1087074

**Published:** 2022-12-22

**Authors:** Arun K. Bhunia, Atul K. Singh, Kyle Parker, Bruce M. Applegate

**Affiliations:** ^1^ Molecular Food Microbiology Laboratory, Department of Food Science, Purdue University, West Lafayette, IN, United States; ^2^ Purdue University, Purdue University Interdisciplinary Life Science Program (PULSe), West Lafayette, IN, United States; ^3^ Purdue Institute of Inflammation, Immunology and Infectious Disease, Purdue University, West Lafayette, IN, United States; ^4^ Department of Comparative Pathobiology, Purdue University, West Lafayette, IN, United States; ^5^ Clear Labs, San Carlos, CA, United States; ^6^ Department of Biological Sciences, Purdue University, West Lafayette, IN, United States

**Keywords:** bacterial colony, Petri-plate, optical sensor, laser, scatter signature, BARDOT, Raman, hyperspectral imaging

## Abstract

Classical microbiology has paved the path forward for the development of modern biotechnology and microbial biosensing platforms. Microbial culturing and isolation using the Petri plate revolutionized the field of microbiology. In 1887, Julius Richard Petri invented possibly the most important tool in microbiology, the Petri plate, which continues to have a profound impact not only on reliably isolating, identifying, and studying microorganisms but also manipulating a microbe to study gene expression, virulence properties, antibiotic resistance, and production of drugs, enzymes, and foods. Before the recent advances in gene sequencing, microbial identification for diagnosis relied upon the hierarchal testing of a pure culture isolate. Direct detection and identification of isolated bacterial colonies on a Petri plate with a sensing device has the potential for revolutionizing further development in microbiology including gene sequencing, pathogenicity study, antibiotic susceptibility testing , and for characterizing industrially beneficial traits. An optical scattering sensor designated BARDOT (bacterial rapid detection using optical scattering technology) that uses a red-diode laser, developed at the beginning of the 21^st^ century at Purdue University, some 220 years after the Petri-plate discovery can identify and study bacteria directly on the plate as a diagnostic tool akin to Raman scattering and hyperspectral imaging systems for application in clinical and food microbiology laboratories.

## Introduction

Possibly, the single most important invention in the field of microbiology was the Petri plate, which enabled the separation and isolation of microbes from a complex mixture (**Table 1**). At the time he invented the Petri plate, Julius Richard Petri was a military physician working in Robert Koch’s lab in Germany during the 1880s. Today it is a quintessential tool in microbiology laboratories allowing bacterial isolation, enumeration, mutagenesis, genetic manipulation, antibiotic sensitivity/resistance testing, enzymatic activity, hemolytic activity assessment, and many more (Lagier et al., 2015). Before Petri’s invention, scientists used sliced potatoes’ surfaces as a means for the separation and isolation of microbes, but the approach did not reliably yield a sterile environment. The Petri plate, with a sterile media and a lid, can also be sterilized, providing a pristine environment in which microbes can be cultured, isolated, identified, and studied based on individual colony formation.

**Table 1:** Petri plate and microbiology milestones.

Year	Milestone
1882	Agar replaced gelatin in solid media
1884	The modern autoclave was invented
1887	Dr. Julius Petri invented the Petri plate
1889	An enrichment medium is used to isolate Rhizobium bacteria
1905	MacConkey Agar invented
1907	The presence of the *lac* operon is first observed
1915	Discovery of bacteriophage
1919	Blood is added to agar as a nutrient but also as a differentiating agent
1921	Changes in strain virulence are associated with environmental conditions
1923	D.H. Bergey published the first manual on microbial identification
1928	Transformation in bacteria was discovered
1929	Alexander Fleming discovered penicillin
1940	Agar disc diffusion test for antimicrobial activity testing was developed
1944	The first antibiotic (colicin) for Gram-negative bacteria is discovered
1944	Discovery of DNA as hereditary materials
1946	Bacterial mating was discovered
1957	The technique of replica plating is discovered
1958	Modern streak plating invented
1960	The model for the *lac* operon is developed. Bacteria are shown to have a mechanism where an environmental cue can turn off a gene
1971	Restriction enzymes were discovered.
1975	Colony hybridization
1995	The first bacterial genome was sequenced, *Haemophilus influenzae*
2007	A label-free laser-based detection technique, BARDOT was invented to directly identify a bacterial colony on a Petri plate without destruction further aiding in gene sequencing, pathogenicity study, antibiotic susceptibility testing, and characterizing industrially beneficial traits.

A single bacterial cell divides through binary fission, first in two-dimensional space and subsequently in three-dimensional space (Su et al., 2012), forming a colony on the agar surface. Depending on the organism, colony architecture, shape, size, and chromogen production become characteristic features of a given bacterium for visual identification. A bacterial colony is viewed as a self-engineered multicellular organism exhibiting intricate communication skills and social intelligence (Ben Jacob et al., 2004; Ben-Jacob and Levine, 2006), in response to the adjoining environment (Ben-Jacob et al., 1998; Shapiro, 1998; Dunny et al., 2008). Genotypic variations in an individual bacterium amplify in the colony, by a million or billion-fold, which affects the colony morphotype (Ben-Jacob et al., 1998; Brehm-Stecher and Johnson, 2004).

**Figure 1:**
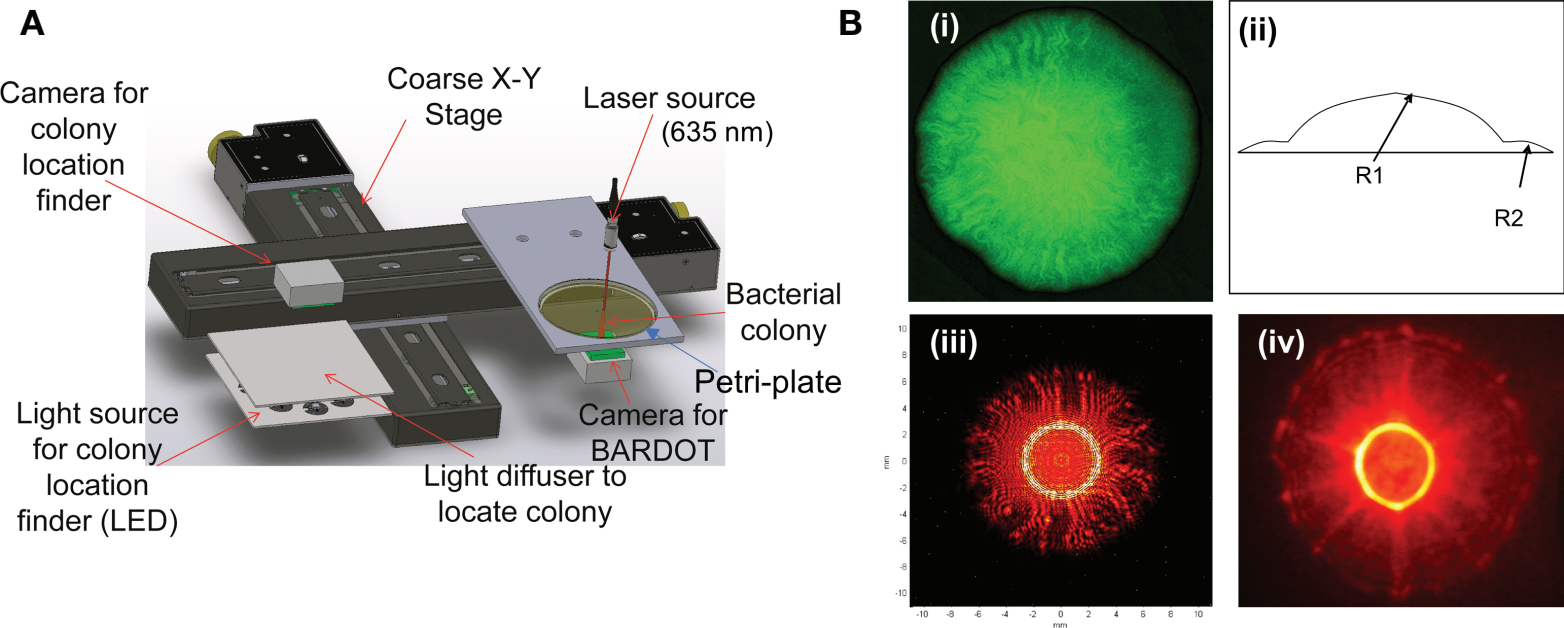
**(A)** Schematics of BARDOT (Bacterial Rapid Detection using Optical scattering Technology). Source (Singh et al., 2014); **(B)** Modeling of BARDOT-generated scatter signature of green-fluorescence protein (GFP) expressing *Listeria monocytogenes* (Lm) colony. (i) Confocal image of the colony (~1 mm diameter) of GFP-expressing Lm, (ii, iii) Scatter image modeling using two-stage curvature R1 and R2, and (iv) actual scatter image of the colony. Source (Banada et al., 2007).

Modern-day nano-bio sensors have begun to revolutionize the detection of a single molecule or a single cell with high precision, but most depend on probes (labels) or signature tags for the identification of targets (Bhunia, 2014; Zhang et al., 2016; Ali et al., 2020; Nayl et al., 2020; Xu et al., 2021; Al-Hindi et al., 2022). Very few biosensors operate independently of molecular probes, but are reliant on a database, and are categorized as label-free. We have discovered that a red diode laser (635 nm) upon shining on the center of a bacterial colony generates a unique scatter signature, allowing direct and instantaneous identification of bacteria on the Petri plate without disturbing the colony integrity (Banada et al., 2007) (**Figure 1**). The device is called BARDOT (Bacterial Rapid Detection using Optical scattering Technology), which exploits genetic (metabolic) and phenotypic (functional) differences in microbes for identification and can aid in screening the most desirable isolates for further study including gene sequencing, pathogenicity, antibiotic susceptibility, vaccine development, and for characterizing industrially beneficial traits (Bhunia, 2014; Miller et al., 2020; Xu et al., 2021).

Recent emphasis on culturomics (Fournier et al., 2013) lead to explosive progress in the development of specialized growth media for the isolation of microbial pathogens on Petri-plate. Integration of culturomics with the biophysical identification tool such as BARDOT would not only help in making an informed decision but also would be an indispensable tool for early diagnosis and detection of microbes in human and veterinary medicine, food hygiene, and agriculture (plant and fish pathogens). Petri plate together with the BARDOT device would aid in high throughput screening of a large number of colonies for identification and facilitate polymerase chain reaction (PCR)-based confirmation, whole-genome sequencing, and mass spectrometry (Bhunia, 2014). BARDOT can also be used to study pathogens and spoilage microbes and the microbial community and their shift in response to environmental cues. In this review, the historical perspectives of the Petri plate and its contribution to the modern-day microbial study using laser optical sensors, including BARDOT, Raman, and hyperspectral imaging systems are emphasized.

## History of the “Golden Age” of microbiology

### Petri plate

“We have here the first description of the Petri dish, a simple yet effective device for culturing microorganisms on solid media” – R.J. Petri, 1887 (Petri, 1887). Julius Richard Petri, a military physician working in Robert Koch’s lab in Germany during the 1880s invented the Petri plate. In 1887, Petri was trying to find a solution to the problem of culture contamination. Before the development of the Petri plate, scientists used various growth media that would allow their cultures to grow, such as sliced potato, cooked egg whites, and gelatin. These were all attempts at creating a solid growth medium that would facilitate the growth of microorganisms. To make the sample contaminant-free, a bell jar was placed over the culture. Petri’s innovation created a simple tool that effectively provides a small, isolated environment for a culture to grow free of contamination.

The modern Petri plate was the culmination of several innovations that created a sterile environment for bacterial growth. In 1882, Frannie Hesse, the wife and lab technician of Walter Hesse, began substituting agar for the gelatin that she was putting into his solid media in tubes. By replacing gelatin with agar, scientists were able to have a stable solid media that could be incubated, could not easily be degraded by microbes, and was transparent. The invention of the autoclave by Charles Chamerbland in 1884 allowed for the complete sterilization of equipment and media. In 1887, when Julius Petri developed a glass plate that had another, larger plate as its lid, he created an intuitive tool that was compact, reusable, and able to house any solid media. With the combination of these three technologies, the modern Petri plate was born. Scientists could now be certain that their cultures were stable due to their agar base, sterile because of the use of the autoclave, and safe from contamination. This simple solution was fundamental to the primary use of solid media for the isolation and separation of culturable bacteria. His plates have since allowed researchers to easily isolate, observe, study, and manipulate the microorganisms. The primary function of routinely separating and isolating microbes on solid media can not be trivialized for its impact as a routine and integral part of traditional or modern microbiology laboratories. Its use has affected and still impacts all aspects of our lives. Some primary examples can be seen in food processing for evaluating the efficacy of microbial inactivation methods to eliminate pathogens and spoilage organisms and biotechnology for isolating recombinant organisms expressing foreign proteins for medical and commercial applications. An example of the Petri plate’s importance in research can be illustrated in its use in the transformative publication “Studies on the chemical nature of the substance inducing transformation of pneumococcal types” (Avery et al., 1944). The Petri plate was the tool used to determine that DNA was the genetic material by examining the rough and smooth phenotypes of *Streptococcus pneumoniae* after growth on plates.

The differences in phenotypes in Avery et al.’s work (Avery et al., 1944) were obvious and visualized with the naked eye. However, if a more detailed nondestructive analysis of bacterial cultures on plates could be obtained to detect differences in genus, species, and even strains of bacteria it would be of great value to the microbiology community. In 2007, our lab at Purdue University reported a prototype laser optical sensor, called BARDOT (bacterial rapid detection using optical scattering technology) that is capable of bacterial colony detection and characterization directly on the agar surface of the Petri plate using a red-diode laser beam (Bae et al., 2007; Banada et al., 2007) culminated from the earlier groundbreaking work using a laser to physically map bacterial cell morphology on a Petri plate (Nebeker et al., 2001).

## Direct method of detection and identification of bacterial colonies by light scattering sensor

### BARDOT

Laser-based interrogation of individual bacterial cells in liquid suspension has been previously attempted by P.J. Wyatt and his team in the late ‘60s (Wyatt, 1968; Wyatt, 1969). Measuring the intensities of the full 4-π radian of scattered light from single cells was the detection principle where the light scattering phenomenon occurs in a single scattering regime. The system requires the suspension of one type of organism (purified target culture) to avoid the generation of multiple overlapping scatter signatures from mixed cultures since multiple scattering events may interfere with the specific detection of the target (Haavig et al., 2017). Therefore, pure cultures are essential, which could be obtained from isolated colonies from a Petri plate. Furthermore, such a detection approach also requires a very low cell density (about 100 cells/ml) to ensure capturing of the single scattering event and avoiding interferences from other scatterers.

Our approach in using elastic light scattering for bacterial interrogation was focused on bacterial microcolonies (0.7 mm to 1.2 mm diameter). Single bacterial cells, through binary fission, give rise to a colony on an agar surface, providing masses of cells that can provide a volume of scattered light for the interrogation of those bacteria with relative ease. In BARDOT, a laser beam with 635 nm (1 mm diameter) wavelength and 1 mW power is passed through the center of spatially located well-separated bacterial colonies (about 1 mm diameter) on a Petri dish and generates a unique scatter signature with circular boundaries with concentric rings, radial spokes, wavy lines, or speckles as a fingerprint. Each scatter pattern is unique for a bacterial culture at the species and serovar level on an agar plate containing specific growth media at a specified time of growth (Banada et al., 2009; Bae et al., 2011a). Colony scatter patterns changes with time; therefore, it is also critical to find a time window when a scatter signature with multiple features can be reliably used for bacterial identification (Bae et al., 2008; Banada et al., 2009). The physics behind the forward scattering is well understood (**Figure 1**): An incoming wavefront is capable of interacting with the micro/macro structures of a colony which ‘imprints’ its signature on the outgoing wavefront. This is further propagated to the detector and decodes the characteristics of scattered light intensity (Bae et al., 2007; Banada et al., 2007; Bae et al., 2010).

The BARDOT unit is designed with two subcomponents: a microbial colony locator and a forward scatterometer. The first component is responsible for counting and locating the center coordinates of the individual colony and excluding those that don’t match the detection criteria (doublets or diameter outside of the detection range). The colony locator consists of a ring-type light-emitting diode (LED) array for illumination purposes along with the plate diffuser to provide equal illumination across the plate. A monochromatic CMOS (complementary metal-oxide-semiconductor) camera with 1024 x1280 pixels is located on the top of the plate along with an imaging lens with a viewing angle of 34° x 25.6°. Once the candidate colonies for detection are determined, the list of center locations for those colonies is sent to the forward scatterometer. This subcomponent is responsible for capturing the forward scatter patterns from each colony and the scatter features are captured with a second CCD (charge-coupled device) camera placed on the bottom of the Petri dish. To ensure the capture of a quality scatter pattern, a centering algorithm that minimizes the distance between the center of the laser and the colony has been implemented by calculating the difference in geometric moments (**Figure 1**).

A four-quadrant balancing algorithm was used for quantitatively aligning the laser with the colony while a “traveling salesman algorithm” was implemented to minimize the traveling time between two colonies (Bae et al., 2009). At the same time, automated image processing and classification software were also integrated for seamless analysis and identification of microbes for high throughput screening (Rajwa et al., 2010).

Using the same optical scattering principle, Buzalewicz et al. (Buzalewicz et al., 2019) reported the development of BISLD (Bacteria Identification System by Light Diffraction) for the detection of variable-size colonies at a fixed incubation period by adjusting laser beam diameter. A similar fixed incubation approach using the laser scattering method was also employed by others to interrogate variable-size bacterial colonies on a Petri dish (Marcoux et al., 2014; Minoni et al., 2015). Such an approach can overcome the major limitation of BARDOT which uses a fixed-diameter laser beam targeting colonies of a specific size range (0.7 mm – 1.2 mm) while ignoring the colonies outside this range.

### BARDOT generated scatter image analysis and pathogen identification

Once the scattering patterns are captured, they are stored in the database as a fingerprint library for future detection and presumptive identification of the bacteria using advanced classification algorithms. The captured scatter patterns of the bacterial colony are automatically analyzed by the quantitative image processing software. Two major features are used for image analysis: the rotation-invariant feature (circularly symmetric patterns) and texture features (random and speckle patterns). The performance of the classifier is estimated using cross-validation (Baldi et al., 2000). The importance of quantitative classification software is needed to reduce human errors, which in turn can provide higher sensitivity and specificity than visual observation. Each 2-D scatter pattern is analyzed *via* Zernike moments and Haralick textures (Bayraktar et al., 2006). The former extracts features of circularly symmetric features from the scatter patterns, while the Haralick describes the texture of the scatter patterns. The combination of these two features results in hundreds of signature attributes from a single 2-D image which can be used as an orthogonal basis for the fingerprint library in the classification of scattering patterns of bacterial test samples.

Once the training library is built, the sample under investigation can be compared against the fingerprint library that is already built and trained. The results are then reported in a matrix format. The diagonal numbers represent the expected correct classification rate (true positive and true negative) while the off-diagonal numbers show the missed classification (false positive and false negative). More details about the CV matrix are discussed in our relevant publications (Banada et al., 2009; Rajwa et al., 2010).

### Raman spectroscopy

Raman spectroscopy uses a laser to record the vibrational and rotational properties of molecules yielding a scattering signature referred to inelastic scattering (Petry et al., 2003; Stöckel et al., 2016). Earlier attempts to detect microcolonies of clinically relevant bacterial pathogens of *Staphylococcus aureus*, *Staphylococcus epidermidis*, *Escherichia coli* and *Enterococcus faecium* directly from agar plate using a Raman microspectrometer equipped with an 830-nm titanium−sapphire laser at wavenumber 250 to 2150 cm^-1^ was moderately successful (Maquelin et al., 2000). Later, Rosch et al. (Rösch et al., 2003) used lasers with three different wavelengths (785 nm; 633, and 514 nm) to differentiate colonies of test organisms (*Micrococcus luteus*, *Bacillus subtilis*, and *Pseudomonas fluorescens*) where chromophores produced by these organisms aided in spectral classification. However, bacterial viability was lost due to the destruction of bacterial cells during laser exposure, a major impediment to the isolation of viable cells after Raman spectroscopy (Yuan et al., 2018). Raman spectroscopy was also successfully used for the detection of colonies of clinically relevant *Staphylococcus epidermidis, S. aureus* and *Escherichia coli* strains on blood agar or Mueller-Hinton agar plates (Almarashi et al., 2012; Rebrošová et al., 2017b). Most recently, Shen et al. (Shen et al., 2022) reported a rapid fiber probe-based Raman (785 nm diode laser) technique for the classification and identification of 33 strains of 8 different species including *Candida albicans*, *Staphylococcus epidermidis*, *S. aureus*, *Klebsiella pneumoniae*, *K. oxytoca*, *Escherichia coli*, *Enterococcus faecalis*, *E. faecium*, and *Acinetobacter baumannii* on Luria–Bertani (LB) agar plates. Nevertheless, Raman spectroscopy continued to be an attractive on-plate microbial pathogen detection tool for foodborne (Huayhongthong et al., 2019), and clinical relevant pathogens (Rebrošová et al., 2017a; Nakar et al., 2022). However, bacterial physiological state, growth phase and growth media can affect the spectral fingerprints thus these parameters should be controlled with care (Assaf et al., 2014; Mlynáriková et al., 2015).

### Hyperspectral imaging

Hyperspectral imaging (HSI) technology combines spectroscopy and imaging as a reliable nondestructive technique for bacterial colony counting, and detection and identification on various food, inert surfaces, or clinical specimens (Gowen et al., 2015; Park et al., 2015; Bonah et al., 2019; Lu et al., 2020; Soni et al., 2022). Lights with small wavelength bandwidth from visible to near-infrared (Vis-NIR) are often used to generate a complete spatiospectral map of a colony for pathogen detection and identification. Scientists at the US Department of Agriculture developed a hyperspectral imaging system with a spectral range from 400 to 1000 nm to detect and differentiate serovars of Shiga-toxin-producing *Escherichia coli* (STEC) pathogens on Rainbow agar with very high accuracy (Windham et al., 2012; Yoon et al., 2015). They also successfully used this platform to detect *Campylobacter* species (Yoon et al., 2009).

Direct identification of colonies on culture plates is also highly important for clinical diagnostic applications. Arrigoni et al. (Arrigoni et al., 2017) applied HSI coupled with a classification algorithm to identify pathogens that are responsible for urinary tract infection, including *Escherichia coli*, *Enterococcus faecalis*, *Staphylococcus aureus*, *Proteus mirabilis*, *Proteus vulgaris*, *Klebsiella pneumoniae*, and *Pseudomonas aeruginosa* on blood agar plates. HSI was also used for the discrimination of colonies of three different bacterial cultures including *E. coli*, *Listeria monocytogenes* and *Staphylococcus aureus* for application in food safety (Feng et al., 2018). This method employed a non-selective agar plate (tryptic soy agar, TSA) for colony identification. Likewise, using HSI and chemometric classification algorithms, Gu et al. (Gu et al., 2020) differentially distinguished colonies of *Escherichia coli, Staphylococcus aureus*, and *Salmonella enterica* on three different nonselective agar plates. However, the drawback of using non-selective agar plates for HSI application is that the growth of commensal bacteria can interfere with target organism identification when testing with food samples, thus conventional broth culturing techniques using selective antimicrobial agents must be employed before testing food samples on agar plates. Near-infrared (NIR) HSI with multivariate data analysis was shown to be useful for the discrimination of colonies of *Bacillus cereus*, *Escherichia coli*, *Salmonella enterica* serovar Enteritidis, *Staphylococcus aureus* and *S. epidermidis* (Kammies et al., 2016). The application of HSI in a reflectance mode is highly useful for differentially distinguishing bacterial colonies from particulate foods on agar plate surfaces for food safety analysis (Shi et al., 2019).

## Indirect methods of bacterial colony detection and identification

### Mass-spectrometry

As a routine microbiological laboratory practice, bacterial cells collected from a well-separated colony (to assure pure culture) from a Petri dish, are tested for their unique sugar or amino acid utilization patterns as an identifying tool (Bhunia, 2014; Vasavada et al., 2020). Likewise, bacterial cells from colonies are also tested by using the matrix-assisted laser desorption ionization-time of flight (MALDI-TOF) mass spectrometry. A laser beam ionizes the sample matrix creating single protonated ions from analytes in the sample. Using acceleration at a stable potential, protonated ions are separated based on their mass-to-charge ratio. The time of flight measures the mass-to-charge ratio by the time it takes the ion to travel the length of the flight tube (Singhal et al., 2015). The spectral signatures are matched with the database for identification. This method is reliably used for pathogen detection from food and clinical samples (Feucherolles et al., 2019; Nomura et al., 2020).

### Spectroscopy

Inelastic scattering technologies such as near-infrared (NIR) (Dubois et al., 2005), Fourier transform Infrared (FT-IR) (Naumann et al., 1991), Raman (Stöckel et al., 2016), and hyperspectral imaging (Soni et al., 2022) have been used to identify bacterial pathogens that are obtained from isolated colonies from a Petri-dish. Often colony isolated bacterial cell suspensions are dispersed on appropriate substrates (for example, silicon wafer, CaF_2_) and applied to above mentioned inelastic/vibrational spectroscopy for pathogen identification. Michael et al. (Michael et al., 2019) applied HSI to identify several bacterial pathogens including *Cronobacter sakazakii*, *Salmonella* spp., *Escherichia coli*, *Listeria monocytogenes* and *Staphylococcus aureus* smeared on glass slides obtained from an isolated colony from agar plates. Likewise, Raman (Craig et al., 2013; Dina et al., 2017; Liu et al., 2021; Rousseau et al., 2021; Wang et al., 2021; Yan et al., 2021) and FT-IR (Davis and Mauer, 2010; Zarnowiec et al., 2015; Lasch et al., 2018; Vogt et al., 2019) spectrocopies have been shown to be very promising diagnostic tools for detection, identification or antibiotic susceptibility testing of various pathogens obtained from isolated colonies.

In addition, laser-induced breakdown spectroscopy (LIBS) was developed in response to the rapid identification of biothreat agents including pathogens or toxic gas. In this technique, the breakdown of the target analyte by a laser shot (1 ms) reaching a temperature of >10,000 K can generate plasma composed of ionic and atomic species (Morel et al., 2003). Quantitative spectrochemical analyses of plasma allow rapid identification of a target analyte. LIBS have been used for the differentiation and classification of foodborne and clinically relevant microbial pathogens obtained from isolated colonies (Multari et al., 2013; Singh et al., 2018; Rehse, 2019).

### Molecular methods

Molecular methods are also increasingly becoming integral to the pathogen detection regimen in agriculture, food, and medicine. Historically, the colony hybridization technique has been a quintessential tool for verification of the acquisition of a target gene(s) by host microbes, where colonies are transferred from a Petri dish to a membrane for hybridization with a pre-labeled nucleic acid probe (Grunstein and Hogness, 1975). Membrane-transferred colonies are also probed with antibodies (colony immunoblot) for the detection and identification of many pathogens including *E. coli* O157:H7 (Meng et al., 1996), *L. monocytogenes* (Bhunia et al., 1992; Carroll et al., 2000), *Helicobacter pylori* (Rojas-Rengifo et al., 2015) and *Campylobacter* species (Huang et al., 2020a). Polymerase chain reaction (PCR) assay has been also applied to colonies (Colony PCR) for the detection of various bacterial (Zimmermann et al., 2014; Daramroodi et al., 2018) and fungal (Lau et al., 2008; Walch et al., 2016) pathogens. Recently, whole genome sequencing (WGS) of isolated colonies is also finding widespread application in food safety and clinical medicine (Hasman et al., 2014; Joensen et al., 2014; Köser et al., 2014; Purushothaman et al., 2022).

## BARDOT-based pathogen detection approaches for individual pathogens

According to the official detection scheme for a pathogen from food or environmental samples, initial liquid culturing in primary and/or secondary enrichment broths followed by plating of the enriched samples on various selective agar plates for isolation of individual colonies are practiced for presumptive identification (Bhunia, 2014). Enrichment broths help resuscitate stressed or injured cells to expand and antimicrobial selective agents in broths help reduce background microflora (Bhunia, 2014). To expedite the diagnostic workflow for faster results (less than 24 h), enrichment broths are routinely tested by a pathogen-specific PCR assay or antibody-based lateral flow immunochromatographic assays (Urusov et al., 2019; Huang et al., 2020b; Fogaça et al., 2021). Bacteriophage-based pathogen detection is also gaining significant interest among microbiologists (Al-Hindi et al., 2022). However; most clinical laboratory diagnostic approaches rely on isolated individual colonies, which are achieved by direct plating of clinical samples on Petri plates. Depending on the species of the bacteria to be isolated, differential or selective agar media are used to obtain a colony with typical phenotypic characteristics (color, texture, diameter). The bacterial growth rate on the Petri plate varies widely and may typically require 12 to 48 h to obtain a colony with a 1-2 mm diameter depending on the genus/species, the selective agents used in the agar media, and the physiological state of the bacteria. While extremely slow-growing organism (ex. *Mycobacterium* species) requires several days. Application of BARDOT can significantly shorten the liquid enrichment and on-plate growth time yielding results much earlier than the conventional culturing method (Bhunia, 2014).

**Figure 2:**
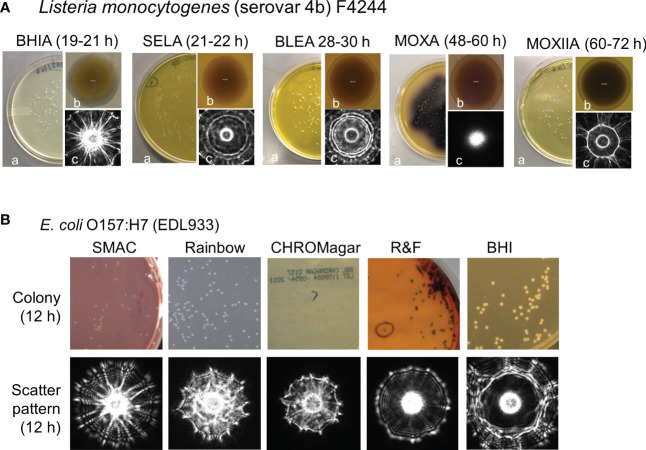
Variation in colony appearance and scatter signatures of (*Listeria monocytogenes* **(A)** and *E*. *coli* O157:H7 **(B)** grown on various growth media: BHIA, brain heart infusion agar; SELA, Salmonella Escherichia Listeria agar; BLEA, Buffered Listeria enrichment agar; MOXA, modified Oxford agar; MOXIIA, modified Oxford agar media without ferric ammonium citrate. a, Petri dish with colonies; b, individual colony magnified 100x; c, corresponding scatter signature (Abdelhaseib et al., 2019). **(B)**: SMAC, sorbitol MacConkey; Rainbow; CROMagar, R&F and BHI were used) (Tang et al., 2014).

As mentioned above, colony isolation is essential for performing more comprehensive tests such as mass-spec analysis, whole-genome sequencing, pathogenicity testing, antibiotic susceptibility analysis, sensitivity to various food preservatives or chemical or biological sanitizers, and other physiological parameters (Bhunia, 2014). On the other hand, BARDOT can be applied directly to the colonies growing on the Petri dishes for interrogation without any physical contact with the colony thus preserving colony integrity and cell viability. The cellular organization, extracellular matrix, phenotype variation, refractive indices, and size of cells within the confinement of a colony are attributed to producing differential scatter signatures (Banada et al., 2007; Banada et al., 2009). Changes in media formulations can also alter scatter signature patterns implying nutrient utilization and metabolic activity are directly linked to a bacterial phenotype which can be used for further validation of cultural identity (Bae et al., 2011a; Abdelhaseib et al., 2019) (**Figure 2**). Further molecular, immunological or biochemical testing of colonies can be done to validate BARDOT results (Xu et al., 2021).

Therefore, the Petri plate is considered to be an irreplaceable tool for both clinical diagnosis and food testing. We will review how BARDOT is utilized for the detection and identification of major pathogens of interest in public health and food safety.

BARDOT has been used to interrogate colonies on Petri plates of various foodborne and clinically relevant bacterial pathogens, such as *Listeria monocytogenes* (Banada et al., 2007; Banada et al., 2009; Abdelhaseib et al., 2019), *Vibrio* species (Huff et al., 2012), *Escherichia coli* O157:H7 (Banada et al., 2009; Tang et al., 2014), *Salmonella enterica* (Singh et al., 2014; Singh et al., 2015a; Abdelhaseib et al., 2019), *Bacillus* spp. (Singh et al., 2015b), *Staphylococcus* spp. (Alsulami et al., 2018), and *Campylobacter* spp. (He et al., 2015) and the members of the *Enterobacteriaceae* family (Singh and Bhunia, 2016). Hence, BARDOT has been considered a non-invasive, non-destructive, and reagent-free detection platform for pathogens of food and clinical relevance. In addition, BARDOT showed utility in differentiating mutant strains deficient in virulence-gene in *L. monocytogenes* (Singh et al., 2016) or antibiotics-induced stress response by bacterial pathogens (Singh et al., 2015a; Zhu et al., 2018). The optical forward scattering technique was also evaluated for application in clinical microbiology for the detection of colonies of *E. coli*, *S. aureus*, *Proteus mirabilis*, *Yersinia enterocolitica*, and *Salmonella* Typhimurium in an automated pathogen identification platform with the variable as well as fixed incubation time (Minoni et al., 2015).

Elastic light scattering device has also been reported by Kitaoka et al. (Kitaoka et al., 2020) demonstrating the capacity to detect and identify various microorganisms, including *Bacillus subtilis*, *S. aureus*, and *Saccharomyces cerevisiae*. Using the BISLD system with improved Fresnel diffraction pattern analysis, Buzalewicz et al. (Buzalewicz et al., 2021) successfully detected *Candida albicans* and several clinically relevant bacterial species including *Citrobacter freundii, Enterobacter cloacae, Enterococcus faecalis, E. faecium, Escherichia coli, Klebsiella oxytoca, K. pneumoniae, Pseudomonas aeruginosa, P. putida, Serratia marcescens, *and* Staphylococcus aureus* with 97-100% accuracy.

### *Listeria* species

*Listeria monocytogenes* is an opportunistic invasive pathogen and is the primary pathogenic member of the genus which has over 27 species. Food is the primary vehicle for transmission. *L. monocytogenes* infects immunocompromised individuals such as the elderly, neonates, and pregnant women resulting in premature birth or stillbirth and the case fatality rate is about 19%. Depending on the official scheme as outlined by USDA-FSIS, FDA, or ISO methods (Barre et al., 2016) for *Listeria* from food or environmental samples, detection involves culturing in primary (ex. UVM, University of Vermont Medium) and/or secondary enrichment broths (ex. Fraser broth) followed by plating on various selective agar plates (ex. Modified Oxford agar, MOX) for isolation of individual colonies exhibiting typical colony morphology that aid in presumptive identification. PCR and lateral flow immunoassays are used for the detection of *Listeria* from enrichment broths for faster results and to bypass the lengthy plating and colony isolation steps (Bhunia, 2014; Fogaça et al., 2021; Lopes-Luz et al., 2021; Xu et al., 2021).

For the application of BARDOT in *Listeria* detection, the laser beam was directly applied to the microcolonies (about 1 mm diameter) on agar plates (MOX prepared without ferric ammonium citrate to prevent the black precipitate formation at the center of the colony or BHI plates) to demonstrate the feasibility of differentiating species of *Listeria* within the genus based on their scatter patterns (Banada et al., 2007; Banada et al., 2009). Image analysis software using Zernike moment invariants and principal component analysis subjected 91–100% accuracy in detecting different species of *Listeria*. Diffraction theory was used to model the scattering patterns to explain the appearance of radial spokes and the rings seen in the scattering images of *L. monocytogenes* (**Figure 1** and **Figure 2**).

In another study, Kim et al. (Kim et al., 2015) employed BARDOT as a complementary tool to differentiate between certain species of *Listeria sensu stricto* and *Listeria sensu lato* (Orsi and Wiedmann, 2016) using *Listeria* species-specific PCR assays. PCR assay amplified a housekeeping gene (*lmo1634*) encoding acetaldehyde alcohol dehydrogenase (AdhE), also known as *Listeria* adhesion protein (LAP) (Drolia et al., 2018). Both PCR and BARDOT were complementary in their abilities to detect *Listeria* from inoculated food samples that contained mixed *Listeria* cultures with a detection limit of about 10^4^ CFU/mL.

In two separate studies, Koo et al. (Koo et al., 2011) and Mendonca et al. (Mendonca et al., 2012) used BARDOT to confirm the presence of *L. monocytogenes* in food samples. They used antibody- or receptor-coated magnetic beads to capture *L. monocytogenes* from enriched food samples before plating them onto selective agar plates. Colonies from Petri plates were analyzed by BARDOT for confirmation. More recently, Zhu et al. (Zhu et al., 2020) used BARDOT to detect *L. monocytogenes* from inoculated milk samples. Application of BARDOT to detect *L. monocytogenes* from experimentally infected mouse tissues was also demonstrated (Banada et al., 2009) where, *L. monocytogenes* was successfully detected in liver, spleen, and intestinal chymus demonstrating the feasibility of BARDOT in potential clinical diagnostics.

### Salmonella enterica

*Salmonella enterica* causes typhoid fever and gastroenteritis. It is estimated that each year in the United States, among 9.4 million foodborne illnesses, gastroenteritis causing non-typhoidal *Salmonella* (NTS) alone is responsible for 1 million illnesses, 19,581 hospitalizations, and 378 deaths (Scallan et al., 2011). *Salmonella* is a robust organism and can survive at low pH, high salt, desiccation, and thermal processing, making it imperative for food companies to develop comprehensive food safety programs to reduce contamination and prevent contaminated products from reaching consumers.

**Figure 3:**
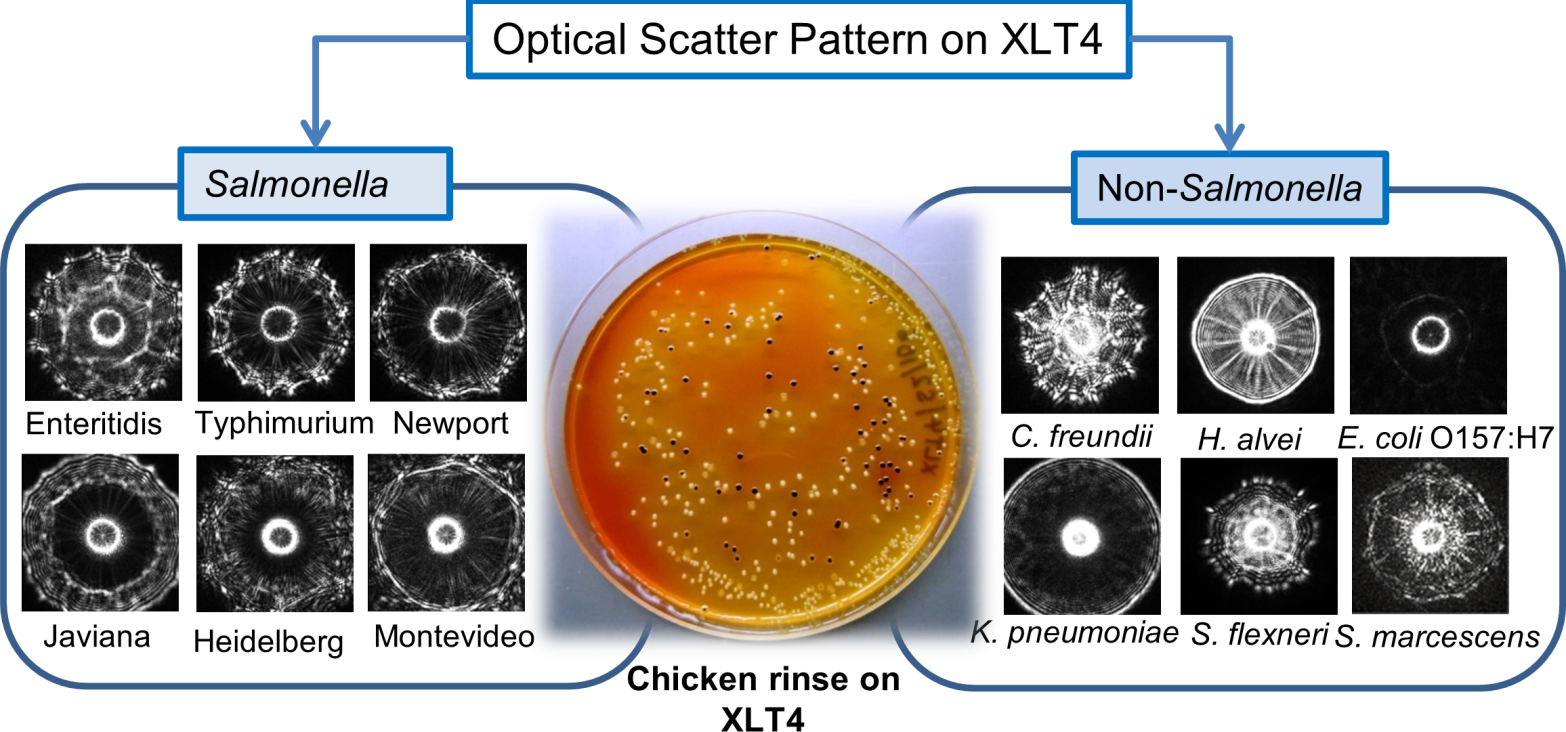
Detection and identification of colonies of *Salmonella enterica* serovars using BARDOT in the presence of background non-*Salmonella* bacteria on Salmonella selective XLT4 agar plate. Source (Singh et al., 2014).

BARDOT was applied to investigate its ability to selectively detect and identify NTS from the top twenty frequently reported serovars of *Salmonella enterica* (Singh et al., 2014). The initial study involved the capacity of BARDOT to classify colonies of six *Salmonella* serovars grown on brain heart infusion (BHI), brilliant green (BG), xylose lysine deoxycholate (XLD), and xylose lysine tergitol 4 (XLT4) agar plates. Cultures on XLT generated highly accurate discriminatory (95.9%) scatter signatures among the *S. enterica* serovars (**Figure 3**). Later, BARDOT yielded classification precision of 88-100% when tested with 36 serovars (top 20 plus 16 miscellaneous serovars), which showed a strong correlation with pulsed-field gel electrophoresis (PFGE)-based genetic fingerprints.

For testing of food samples for *Salmonella* using BARDOT, a sequential enrichment in nonselective (buffered peptone water) and selective enrichment (modified Rappaport Vassiliadis) broths for 4 h each followed by growth on XLT4 (~16 h) was used. BARDOT delivered results within 24 h with a detection sensitivity of 1.2×10^2^ CFU/30 g, much faster than the USDA-FSIS method, which requires about 72 h. Genetic analysis (16S rRNA gene sequencing and PFGE) also confirmed BARDOT results.

In another study, a combination of a fiber optic immunosensor (Valadez et al., 2009) and BARDOT was used to detect *Salmonella* from naturally contaminated poultry samples (Abdelhaseib et al., 2016). Poultry samples were sequentially enriched in primary and secondary enrichment broths for 4 h each before plating on selective agar plates (XLT4). The scatter signatures of colonies on XLT4 generated by BARDOT were matched with the image library for identification of *Salmonella* in less than 24 h. While the fiber optic sensor was applied directly to the broth sample from secondary enrichment thus results were obtained in less than 12 h. Though the fiberoptic sensor provided faster results, the BARDOT-based detection approach provides an opportunity to obtain pure isolated colonies that can be further used for antibiotic sensitivity testing, whole-genome sequencing, source tracking and other studies including pathogenicity assays.

### *Bacillus* species

**Figure 4:**
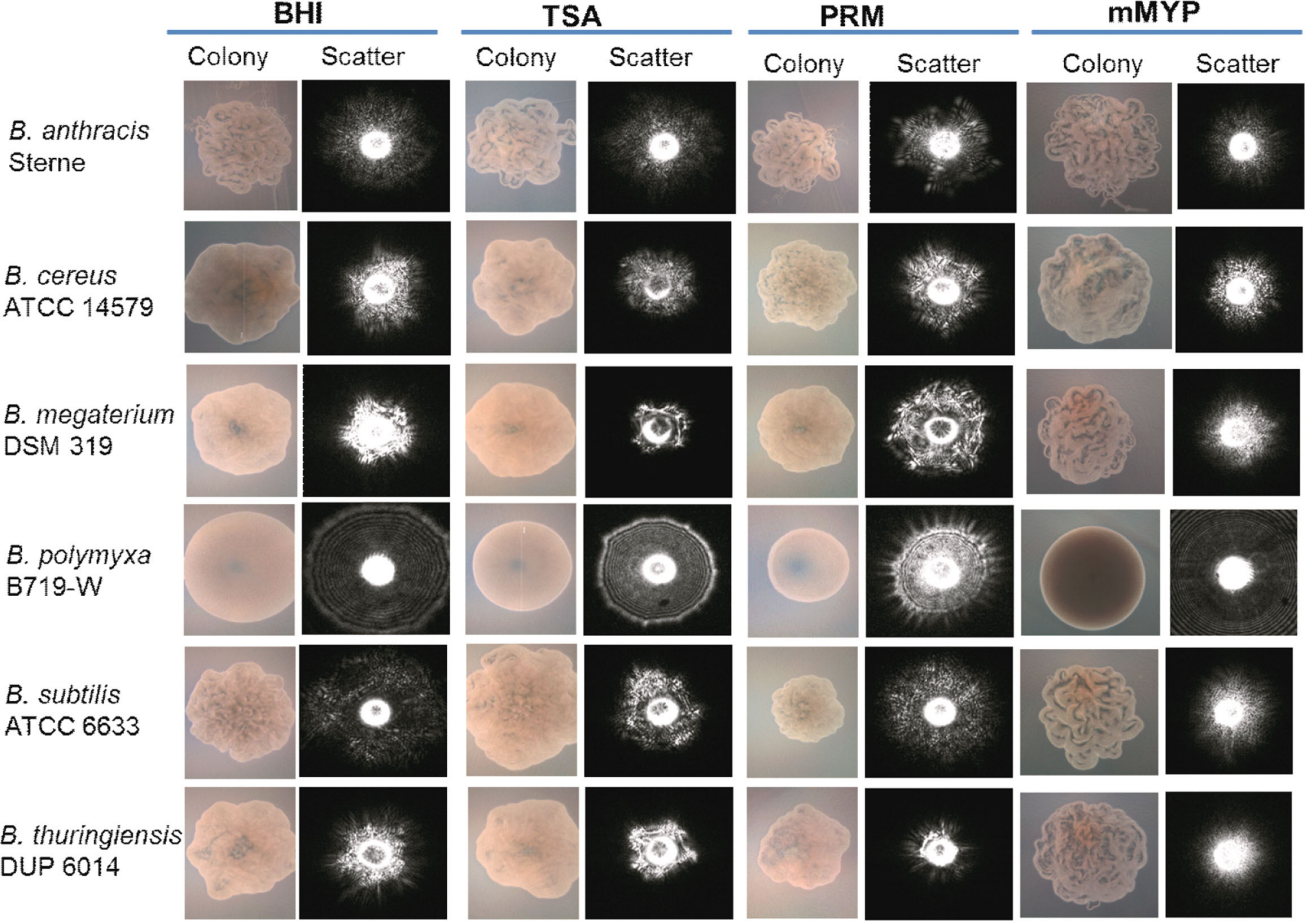
Microscopic images of colonies and corresponding scatter images of colonies of *Bacillus* species grown on BHI, brain heart infusion; TSA, tryptic soy agar; PRM, phenol red mannitol; and mMYP, modified mannitol egg yolk polymyxin without egg yolk. Source (Singh et al., 2015b).

The genus *Bacillus* comprises pathogens, nonpathogens, and industrially relevant beneficial bacteria. Bacilli are spore-forming Gram-positive bacteria. Singh et al. (Singh et al., 2015b), used BARDOT to screen and differentiate colonies of *Bacillus* species on Petri plates containing phenol red mannitol (PRM) agar (**Figure 4**). Colony morphology is highly diverse among the species of *bacillus* producing a flat surface with rough topography therefore the colony scatter patterns consisting of speckles are unique and do not overlap with scatter patterns from other bacterial species (Kim et al., 2014). Initially, a colony scatter image library was created using a total of 265 *Bacillus* and non-*Bacillus* isolates from our collection. Cross-validation experiments demonstrated that all *Bacillus* species (*n* = 118) gave a positive predictive value (PPV) above 90% while non-*Bacillus* spp. showed a PPV of <0.5%.

Spiked baby formula and cheese samples, or naturally contaminated bovine unpasteurized milk samples were surface-plated on PRM and the microcolonies were scanned by BARDOT in 7-16 h to capture colony scatter signatures. BARDOT-identified *Bacillus* cultures were further verified by using PCR and 16S rRNA gene sequencing to provide high accuracy (Singh et al., 2015b).

### Shiga toxin-producing *E. coli*

*Escherichia coli* is one of the ubiquitous Gram-negative bacteria that resides in the intestine of animals and humans. A majority of *E. coli* are nonpathogenic while a small subset is pathogenic and causes diseases including gastroenteritis, urinary tract infection, kidney disease, and central nervous system infection. Based on the nature of the gastrointestinal infection, *E. coli* is grouped into 5 major pathotypes; enterotoxigenic *E. coli* (ETEC), enteroaggregative *E. coli* (EAgEC), enteroinvasive *E. coli* (EIEC), enteropathogenic *E. coli* (EPEC), and enterohaemorrhagic *E. coli* (EHEC), which is a subset of broadly defined Shiga toxin-producing *E. coli* (STEC) (Croxen and Finlay, 2010; Castro et al., 2017). STEC has emerged as an important foodborne pathogen, among which seven serogroups (O26, O45, O103, O111, O121, O145, O157) are most frequently implicated in human infection. Tang et al. (Tang et al., 2014) used BARDOT to differentiate STEC serovars. The goal was to determine if BARDOT can be used to rapidly identify the colonies of STEC serogroups on selective agar plates. Multiple selective/differential agar media on the Petri plate was evaluated that including sorbitol MacConkey (SMAC), Rainbow^®^ Agar O157, BBL™ CHROMagarO157, and R&F^®^ *E. coli* O157:H7, and BHI (**Figure 2**). Colony scatter signatures obtained after 10-12 h growth on both SMAC and Rainbow produced results that successfully differentiated all seven serovars (O26, O45, O103, O111, O121, O145, O157) of STEC with greater than 90% accuracy. Colonies of *E. coli* O157 and O26 serovars in a mixed culture and inoculated food samples (lettuce and ground beef) were accurately identified by BARDOT requiring a sample-to-result in less than 24 h (Tang et al., 2014).

### *Vibrio* species

The genus *Vibrio* consists of three major human pathogens including *Vibrio cholerae*, *V. parahaemolyticus, V. vulnificus* that are associated with water- and seafood-related outbreaks worldwide (Baker-Austin et al., 2018). *V. cholerae* is responsible for cholera, a severe diarrheal disease. *V. parahaemolyticus* and *V. vulnificus* cause gastroenteritis but may also cause fatal septicemic disease and are primarily transmitted *via* seafood. Their primary habitat is an aquatic and marine environment with a high preference for brackish (salt and fresh water mix) water. These halophiles (salt-loving) organisms also undergo a viable but nonculturable state under stressful conditions thus their culturing becomes very difficult. Culture enrichment in alkaline peptone water can help resuscitate a culturable state. For isolation of individual colonies, the enriched samples can be plated on BHI containing 1% NaCl or on selective Thiosulphate Citrate Bile Salts Sucrose (TCBS) agar. BARDOT was applied for *Vibrio* detection on Petri plates after 12 h growth at 30°C and it successfully detected *V. cholerae*, *V. parahaemolyticus*, and *V. vulnificus* present in oyster or water samples in 18 h even in the presence of other vibrios or other bacteria, indicating the suitability of the sensor as a powerful screening tool for pathogens on agar plates (Huff et al., 2012).

### Staphylococcus aureus

*Staphylococcus* aureus is a major pathogen responsible for nosocomial infections and foodborne illnesses (Cheung et al., 2021). Common habitat for *S. aureus* is nares and skin. BARDOT was used for rapid colony screening and detection of *Staphylococcus* on an agar plate and to differentiate these colonies from non-*Staphylococcus* spp. (Alsulami et al., 2018). Phenol red mannitol agar (PRMA) was used for building the *Staphylococcus* species scatter image libraries since this medium generated distinguishing scatter signatures when compared with other species. The scatter image library for *Staphylococcus* species gave a high positive predictive value (PPV 87.5–100%) when tested against known laboratory strains of *Staphylococcus* spp., while the PPV against non-*Staphylococcus* spp. was 0–38%. BARDOT detected *S. aureus* with 80–100% PPV from naturally contaminated cow milk and ready-to-eat chicken salad samples and the results were validated with PCR and 16S rRNA gene sequencing (Alsulami et al., 2018).

### Enterobacteriaceae

**Figure 5:**
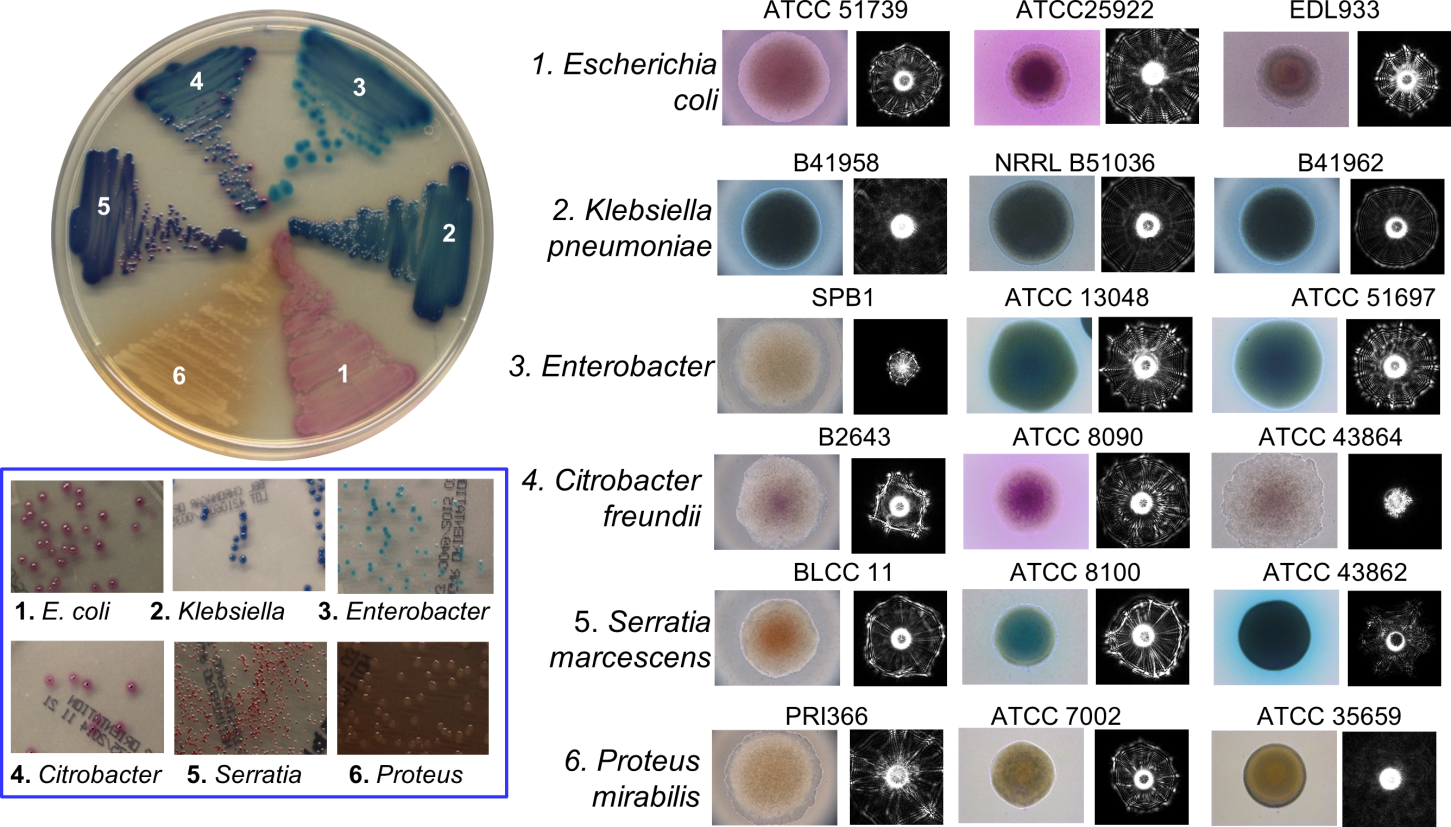
Microscopic images of colonies and corresponding scatter patterns of bacteria from *Enterobacteriaceae* family grown on CHROMagar™ Orientation medium. Source (Singh and Bhunia, 2016).

BARDOT was also used to study colonies formed by the members of the *Enterobacteriaceae* family, which comprises pathogens and commensals and has a significant impact on food safety, clinical microbiology, and public health. Their presence in foods/water indicates potential contamination with pathogens. *Enterobacteriaceae* (EB) detection has been used as an indicator for assessing the safety of food products or water. Various selective chromogenic media are used for quantification and analysis of colonies of EB; however, many produce similar chromogenic by-products thus they cannot be accurately visually identified on the Petri plate. Singh and Bhunia (Singh and Bhunia, 2016) applied BARDOT to screen colonies of the *Enterobacteriaceae* family including *Klebsiella, Enterobacter, Citrobacter, Serratia, Proteus, Morganella*, and *Providencia* cultured on CHROMagar™ Orientation medium (**Figure 5**). A scatter image library (1683 scatter images) was made that contains colony scatter signatures of 36 isolates representing 12 genera and 15 species. This library helped BARDOT-based detection of colonies of members of *Enterobacteriaceae* and non-*Enterobacteriaceae* family (*Pseudomonas aeruginosa*, *Acinetobacter* spp., and *Staphylococcus aureus*) with high accuracy (83-100%) in 10-22 h or even before visible production of chromogens.

### BARDOT as a tool to study pathogenesis, physiology, and community diversity

BARDOT was used in the screening of mutant strains that are deficient in several virulence genes essential for pathogenicity (Singh et al., 2016). During microbial pathogenesis studies, virulence-encoding genes are routinely disrupted by deletion or insertion to create mutant strains. Screening mutant strains is a laborious process involving plating on growth media containing antibiotics marker, replica plating, colony hybridization, DNA isolation, and PCR or immunoassays. BARDOT was used to screen virulence-gene-associated mutant colonies during microbial pathogenesis, co-infection, and genetic manipulation studies in *L. monocytogenes*. BARDOT generated differential scatter patterns in *L. monocytogenes*, deficient in *Listeria* adhesion protein (*lap ^-^*), Internalin A (Δ*inlA*), and an accessory secretory protein (Δ*secA2*). Furthermore, the mutant strains complemented with respective genes were also able to restore the scatter signature to that of the WT (Singh et al., 2016). BARDOT was also useful in differential counting of mutant strains in the presence of WT strain in a co-infection experiment. These data demonstrate that BARDOT can be used as a label-free tool to aid researchers in screening virulence-gene-associated mutant colonies during microbial pathogenesis and genetic manipulation studies.

In another study, Singh et al. (Singh et al., 2015a) also investigated the streptomycin-induced stress response in *Salmonella enterica* serovars with BARDOT. Streptomycin-sensitive or streptomycin-resistant *Salmonella* serovars were exposed to various levels of streptomycin and grown on Petri plates and the colonies were screened by BARDOT to assess their stress responses and colony scatter signatures. A substantial qualitative and quantitative difference in the scatter signatures was observed for colonies that were grown in the presence of streptomycin than the colonies grown in the absence of antibiotics. Levels of a stress response protein, GroEL, were increased in the colony confirmed by mass-spec, quantitative RT-PCR, and immunoassays that were implicated to contribute to the differential scatter patterns. The study highlights the suitability of the BARDOT to investigate stress response in bacteria in conjunction with molecular or other analytical methods.

In another study, Zhu et al. (Zhu et al., 2018) used BARDOT to study the effect of tunicamycin, a cell wall teichoic acid (WTA) synthesis inhibitor on colony morphology and colony scatter patterns. WTA is a major component of the cell wall of Gram-positive bacteria and plays a significant role in physiology, biofilm formation, and pathogenesis (Brown et al., 2013).

**Figure 6:**
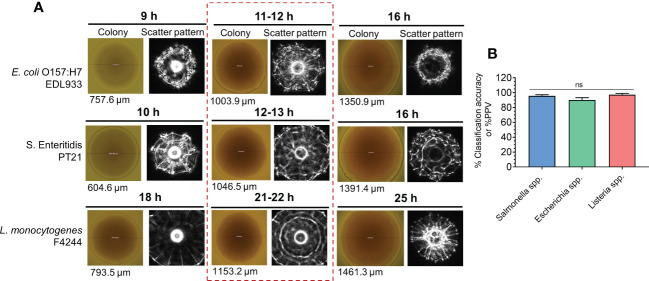
Simultaneous detection and identification of colonies of *E*. *coli* O157:H7, *Salmonella enterica*, and *Listeria monocytogenes* on multi-pathogen selective media, SEL (*Salmonella*, *Escherichia*, *Listeria*) using BARDOT. **(A)** colony scatter signature of colonies after 9-25 h of growth. **(B)** Classification accuracy of all three test pathogens (*E. coli*, *Salmonella* and *Listeria*) after 11-22 h from **(A)** Souce (Abdelhaseib et al., 2019). ns, not significant at P<0.05.

BARDOT was evaluated for its ability to simultaneously detect colonies of three pathogens (*L. monocytogenes*, *Salmonella enterica*, and *E. coli*) from the same test sample, if present together on the same Petri plate (Abdelhaseib et al., 2019). Test samples were first enriched in a multi-pathogen enrichment broth, SEL (*Salmonella*, *Escherichia*, *Listeria*) (Kim and Bhunia, 2008) before plating onto SEL agar for BARDOT-based colony identification. The BARDOT sensor successfully detected *Salmonella*, Shiga-toxin-producing *E. coli*, and *Listeria* on the SEL agar plate with greater than 90% accuracy within 29–40 h demonstrating its simultaneous multi-pathogen detection potential (Abdelhaseib et al., 2019) (**Figure 6**).

BARDOT coupled with 16s RNA sequencing was instrumental in identifying thermostable bacterial colonies in fluid milk subjected to a novel low temperature−short time (LTST) process for pasteurization (Myer et al., 2016).

## Limitations and alternative solutions for colony scattering technologies

The optical forward scattering system, BARDOT for colony detection/identification described here operates based on the propagation of light through the center of the colony and the solid agar media on the Petri plate. Therefore, microbial colonies must be translucent to allow laser propagation for the generation of forward scattering patterns, which is observed for the most of bacterial pathogens tested using BARDOT. In contrast, colonies produced by yeast and mold form opaque structures and thus are unsuitable for interrogation by BARDOT (unpublished observation). Likewise, agar media that are opaque, such as blood agar plate, Baird Parker agar, and media with certain chromogens do not permit laser penetration thus unsuitable for use (Banada et al., 2007; Alsulami et al., 2018). In such situations, a laser-based backscattering device, hyperspectral imaging (Park et al., 2015), or Raman scattering (Stöckel et al., 2016) system can be used to overcome the limitations of forward scattering platforms, such as BARDOT. In addition, variation in agar concentration (above or below the recommended concentration of 1.5% w/w), and media formulations can affect the scatter signatures produced by both elastic or inelastic scatterometers, thus these parameters must be controlled to acquire reproducible scatter signatures (Bae et al., 2009; Banada et al., 2009; Bae et al., 2011a; Assaf et al., 2014; Mlynáriková et al., 2015).

BARDOT-based detection time varies widely since it depends on the rate of bacterial growth to achieve the desired diameter range (0. 7 mm–1.2 mm) that can be detected. Thus fast-growing bacteria can be detected earlier than slow growers. In most cases, as discussed above, a majority of the pathogens were detected in less than 24 h starting with the test sample, providing a culture-based rapid method. BARDOT has been found useful for the detection of physiologically stressed or injured cells, provided a brief resuscitation/enrichment step is included before plating on selective or differential agar plates (Huff et al., 2012; Singh et al., 2015a; Zhu et al., 2018). However, the BARDOT-based method may not be useful for inherently naturally slow growers, such as *Mycobacterium* species, if one is looking to obtain fast results from colony fingerprints.

We also attempted to shorten the BARDOT-based assay time by interrogating microcolonies (0.1–0.2 mm diameter) of three different genera (*Escherichia*, *Salmonella*, and *Listeria* incubated for 7, 9, and 12 h, respectively to achieve 0.1 – 0.2 mm diameter colonies) produced scatter signatures that could be used for differential diagnosis at the earlier time points (Bae et al., 2011b). Similarly, Marcoux et al. (Marcoux et al., 2014) reported similar success in discriminating microcolonies of several Gram-negative bacteria after 6 h of incubation. Overall, microcolony (< 0.2 mm) detection by using scattering technology faces many challenges; (i) precise colony location on the plate due to smaller size, (ii) too small to be differentiated from particulate foods or samples on the plate, and most importantly, (iii) incomplete metabolic activity of growing cells within a microcolony may not produce adequate by-products that can yield robust features to differentiate them from closely related genera or species.

Since the colony scatter patterns of a bacterium depend on a specific growth media, thus for identification, the pathogen and the media-specific scatter image library must be developed for the identification of the target pathogen. Since there are much pathogen-specific selective media available commercially and many more continued to be developed, one must generate an image library database for each medium making the assay development cumbersome and intensive. Most importantly, an image library must be built using bacterial strains that are typed strains procured from authentic and reliable sources (for example, American Type Culture Collection) or genetically validated. In addition, for improved confidence in BARDOT-based identification, the scatter image of the target pathogen could be cross-validated using a scatter image library of commensals that are likely to grow on the selective/differential media (Singh et al., 2014; Abdelhaseib et al., 2016). The use of inelastic scattering technologies (FT-IR, Raman) or molecular methods, such as colony PCR, colony immunoblot, and whole genome sequencing can also be used to validate results.

## Conclusions and future scope

The Petri plate has facilitated some of the most significant discoveries in microbiology. It is important to note that during the beginning of the field of microbiology, many discoveries were made by accident or luck. If Fleming had swept his cultures off the bench into the trash, he may not have seen the zones of inhibition around the *Penicillium* colonies. If he had been using liquid media instead of modern Petri plates, he may not have even noticed the mold contamination. The rise of differential chromogenic or selective media facilitated the discovery of many organisms because it allowed researchers to isolate organisms based on their metabolic activities. Selective, differential, and enrichment media all had a significant impact on the field of microbiology, and the implementation of these media would have been made much more difficult without the Petri plate. Perhaps the single most important effect that the Petri plate has had is its ability to separate, isolate and protect cultures. Not until the modern age of genomic sequencing was a diverse culture able to be analyzed and characterized, hierarchical testing demanded that a pure culture be maintained and tested to characterize and identify the organism. Studies on microbial community and the search for novel probiotics against varieties of ailments would require improved culturing and isolation and Petri-plate would be at the forefront of the exploration. In 2007, researchers at Purdue University reported a light scattering sensor technology, called BARDOT that could scan a diverse sample that had been dispersed on a Petri plate. This technology made it possible to rapidly identify organisms directly from the Petri plate. By directing a red-diode laser (635 nm) through the center of the bacterial colony, BARDOT was able to identify a bacterial colony by its unique scatter image. This high throughput, label-free technology is capable of revolutionizing the way that industries can detect organisms further aiding in gene sequencing, pathogenicity, antibiotic susceptibility, vaccine development, and characterizing industrially beneficial traits. Likewise, the development of hyperspectral imaging and Raman spectrometry tools is gaining significant interest among scientists as reliable on-plate pathogen detection tools. All of this would not be possible without the simple innovation that Julius Petri made in 1887, which we call the Petri plate.

## Author contributions

Conceptualization AB, study design AB, AS, KP, BA, writing and editing AB, AS, KP, BA. All authors contributed to the article and approved the submitted version.
